# Isolation and Molecular Detection of Pigeonpox Virus in a Pigeon With Both Cutaneous and Diphtheritic Forms of Pigeon Pox Disease in Ghana

**DOI:** 10.1155/vmi/7523480

**Published:** 2025-10-26

**Authors:** Richard Kwamena Abbiw, Kingsley Kwabena Amoako, Ben Enyetornye, Theophilus Odoom, Pius Agyenim Boateng, Stacy Magdalene Abbang, Collins Moranga, Tawiah-Yingar Nana Yaw, Patrick Tetteh Ababio, Godwin Dogbey, Richard Suu-Ire, Osbourne Quaye

**Affiliations:** ^1^West African Centre for Cell Biology of Infectious Pathogens, University of Ghana, Legon, Accra, Ghana; ^2^School of Veterinary Medicine, University of Ghana, Legon, Accra, Ghana; ^3^Accra Veterinary Laboratory, Veterinary Service Directorate, Accra, Ghana; ^4^University of Development Studies, Nyankpala, Ghana

**Keywords:** avian poxvirus, cutaneous, diphtheritic, Ghana, pigeon pox virus

## Abstract

Unlike fowl pox, pigeon pox virus (PPV) has not been described in Ghana. This is the first report of the disease and virus in Ghana based on gross necropsy, histopathological analysis, and molecular techniques. This index case was presented as the occurrence of both cutaneous (dry) and diphtheritic (wet) forms of the pigeon pox disease in a 5-month-old female pigeon. Although the observation of Bollinger bodies during histology is confirmatory of pox viruses, the amplification of the p4b gene using polymerase chain reaction and its visualization by gel electrophoresis confirmed the presence of an avian pox virus. Sequencing and phylogenetic analysis of the isolates from this case showed 100% relatedness to the reference PPV isolate FeP2, thus confirming the presence of PPV. The discovery of this virus in Ghana raises the need for surveillance of both wild and domestic avian populations in preparation for any future outbreak, protection of the production birds, and decision on the need for other pox viruses in the country.

## 1. Introduction

Pigeon pox virus (PPV) is a poxvirus in the family *Poxviridae* and genus *Avipoxvirus* [1–4]. The genus, which includes 12 species, is typically classified into three clades (fowl pox virus [FPV], canary pox virus [CPV], and PPV) based on phylogenetic analysis of conserved genes such as *p4b* and DNA polymerase [4]. PPV is a double-stranded enveloped DNA virus causing mild to severe slow-spreading disease in infected pigeons [5–7]. Similar to poxviruses in other species, PPV is generally self-limiting, but can cause more severe disease in young or immunocompromised animals [2].

Transmission of the virus is usually by direct contact through skin abrasions, contaminated feed, water, and eggs [8, 9]. Mosquitoes, other insects, and cannibalism have been implicated in mechanical transmission of the virus [2, 8]. Nonspecific clinical signs include dullness, depression, dehydration, emaciation, and ruffled feathers, and only a few birds develop lesions at a time [10]. PPV leads to the formation of visible wart-like lesions known as pox scabs on mucous membranes and nonfeathered skin [10–13], anorexia, slimy green diarrhea, crouched stance, and rarely neurologic signs including depression [9]. The scabs can be the source of aerosol infection in poultry houses, resulting in respiratory tract infection [2]. The lesions may be described as dry/cutaneous or wet/diphtheritic. The cutaneous form is predominant in most outbreaks and is characterized by scabs on nonfeathered skin, unthriftiness, and reduction in egg production [10–12, 14]. The relatively severe diphtheritic form produces firmly attached, caseous pseudomembranous deposits on and in the mucous membranes of the mouth, tongue, entrance to the trachea, eye, and/or nasal cavity, thereby interfering with feeding or breathing [15]. Gross internal pox lesions do not usually appear in pigeons even though the virus may produce a systemic reaction and occasionally results in a viremia [16, 17]. Latent infection is possible for years with reactivation usually through nonspecific stress factors [4, 14].

Although avian pox has been described worldwide with the exception of Antarctica and Arctic regions [18, 19], reports of PPV in Africa are rare [1, 20–22]. While fowl pox has been documented in Ghana [23] and is relatively common, PPV has not yet been reported [19]. Here, we describe for the first-time gross necropsy, histopathology, and molecular diagnostic findings of PPV in a pigeon (*Columba livia*) in Ghana as a case report.

## 2. Materials and Methods

### 2.1. Case

A 5-month-old female pigeon weighing 0.14 kg kept in quarantine prior to joining 12 other pigeons developed bumps and was presented for veterinary care. All the birds were fed mixed grains of maize, rice, millet, and soya beans. Physical examination revealed multiple nodular lesions of about 0.3–1 cm in diameter. The nodules were unevenly distributed chiefly on nonfeathered areas including around the eyes and the beak. The lethargic bird had droopy and ruffled feathers, greenish stained vent, and was emaciated. The left eye was completely sealed by periocular nodules. The patient was euthanized using cervical dislocation, and a postmortem examination was performed after duly informing the client of the risk to the other birds and success of treatment and seeking the consent of the owner.

### 2.2. Postmortem and Histopathology

Necropsy of the bird was performed as described by Butcher and Miles et al. [24] and Dharanesha et al. [15]. Briefly, the bird was first dipped in water containing disinfectant to reduce the chances of transmission of psittacosis [24] and then examined externally. The internal organs were then regionally examined: the cranial, thoracic, and then abdominal regions. Images of gross lesions were captured and recorded. Nodules on the head and in the oral cavity were sectioned for histopathological processing as described by Dharanesha et al. [15].

### 2.3. Sample Collection and Preparation

Portions of the trachea, feather stalk, esophagus, lung, cutaneous lesions, and gastrointestinal tract were aseptically sampled into sterile tubes and transported on ice to both the Accra Veterinary Laboratory, Veterinary Service Directorate (VSD). Viral inocula were prepared as described by Sultana et al. [5]. Briefly, a 10% (w/v) viral suspension was made by adding phosphate buffered saline (PBS) to the ground samples. The viral suspension was then treated with 300 μL of gentamicin for an hour and cultured on blood agar for 24 h. 0.5 mL of the sterile inocula was injected into embryonated eggs for viral extraction while the rest were stored at −20°C for future use.

### 2.4. Virus Isolation

The virus was propagated by inoculating the prepared inocula into 10–12 days old embryonated chicken hen through the chorioallantoic membrane (CAM) route as described by Rahman et al. [25]. That 0.5 mL of the sterile inocula was inoculated into the center of the CAM using sterile 1 mL, 1.5 inches needle tuberculin syringe, and the opening of the air sac and shell was sealed with melted wax. The inoculated eggs were placed in trays with the CAM upwards and incubated at 37°C for 5 days in an egg incubator. The inoculated eggs were then chilled at 4°C–8°C for 2 h. Afterward, the inoculated eggs were coated with iodine tincture and cracked open, and the thickened CAM was harvested for preparation of inocula as described above (Supporting Figure 1). The viral concentration was increased by performing three passages and the CAMs were collected and transported to the Virology Laboratory at the West African Centre for Cell Biology of Infectious Pathogens (WACCBIP) for further analysis.

### 2.5. DNA Extraction, Polymerase Chain Reaction (PCR), and Gel Electrophoresis

DNA of the virus was extracted using Quick-gDNA Miniprep Kit as per the instruction of the manufacturer. PCR technique targeting pox virus *p4b* gene with the primer sets p2fPF-5′ CAGCAGGTGCTAAACAAACAAA 3′ and p2fPR-5′ CGGTAGCTTAACGCCGAATA 3′ with an amplification size of 578 bp [1, 18] was employed. PCR reaction mixture containing 12.5 μL One Taq 2X master mix, 1 μL each of forward and reverse primers, 6.5 μL nuclease-free water and 4 μL. DNA template was prepared. PCR was done in PCRmax alpha cycler with cycling conditions as follows: initial denaturation at 94°C for 5 min, followed by 35 cycles of denaturation at 94°C for 45 s, annealing at 48°C for 1.5 min, elongation at 60°C for 2 min, and final extension at 60°C for 10 min. Then, a 1.5% agarose gel was prepared, and electrophoresis of the PCR products was done with a follow-up visualization using Amersham Imager 600.

### 2.6. Sequencing and Sequence Analysis

PCR products were purified with Wizard SV Gel and PCR Clean-up System (Promega, USA). Sequencing was performed using MinION (nanopore) sequencing technology and long reads were obtained. Base calling and demultiplexing were performed using guppy basecaller and barcoder, respectively. Quality control was assessed, and samples were trimmed using porechop. The PCR amplified genomic region was aligned to the Reference genome (NC_024447.1; PPV isolate FeP2) and the corresponding region on the reference genome was extracted and blasted using BLASTN algorithm [26] on the standard nucleotide databases (nr/nt). The max-target sequences were 1000 sequences, and a threshold of 0.05 was optimized for highly similar sequences (megablast). Highly identical sequences that looked like duplicates by accession ID or Name or sequence were removed using seqkit, as well as sequences that were not PPV, or were very short sequences. The blast yielded 439 sequences with a percentage identity ranging from 91.43% to 100%, and 111 sequences were removed as they appeared to be duplicated. Out of the remaining 328 sequences 47 were selected based on the above criteria. Sequences (*n* = 47, Supporting 2) were aligned (MAFFT method) and masked at the beginning and end of the alignment, and a phylogenetic tree (RAXML method) was generated using Augur (version Augur-14.0.0) [27].

## 3. Results

### 3.1. Postmortem

**Figure 1:**
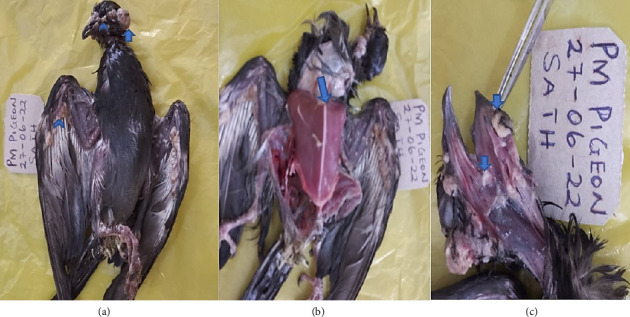
Gross postmortem lesions on the pigeon. (a) Cutaneous nodular lesions of varying sizes. Arrow shows nodules on the eye causing the left eye to be completely shut. Arrowhead shows areas of coalescing of the nodules. (b) Congested carcass with quite prominent keel bone. (c) Diphtheritic nodular lesions in the oral cavity.

Physical examination revealed multiple nodular lesions of about 0.3–1 cm in diameter distributed primarily in nonfeathered regions of the bird (Figure 1(a)), prominent keel bone, and congested breast muscles (Figure 1(b)) and diphtheritic nodules in the oral cavity (Figure 1(c)). Other observed lesions were greenish pasty vent, air sacculitis, and petechial hemorrhages on kidneys which were indicative of possible bacteria coinfections.

### 3.2. Histopathology

**Figure 2:**
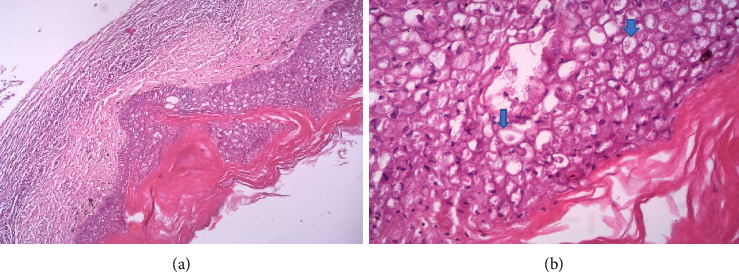
Hyperkeratosis with hyperplasia of the basal epithelium. (a) Epidermis expanded by epithelial cells exhibiting vacuolar/ballooning degeneration. (b) Round eosinophilic intracytoplasmic inclusion bodies (Bollinger bodies).

Infiltrating the epidermis were mixed inflammatory cells (macrophages and heterophils) and areas of necrosis. There is hyperkeratosis with the presence of eosinophilic intracytoplasmic inclusion bodies (Bollinger bodies) in markedly distended and vacuolated keratinocytes with pleomorphic and hyperchromatic nuclei. The presence of Bollinger bodies is an indication that the lesions were caused by a pox virus (Figure 2).

### 3.3. PCR and Gel Electrophoresis

PCR products were observed in all samples except gastrointestinal tract indicating the presence or distribution of pox viruses in the trachea, lungs, cutaneous lesion, feathered stalk, and esophagus. Band sizes of 578 bp were observed. This was congruent with the band size expected for the p4b gene of pox virus (Supporting Figure 2).

### 3.4. Genetic Diversity of PPV in Ghana

**Figure 3:**
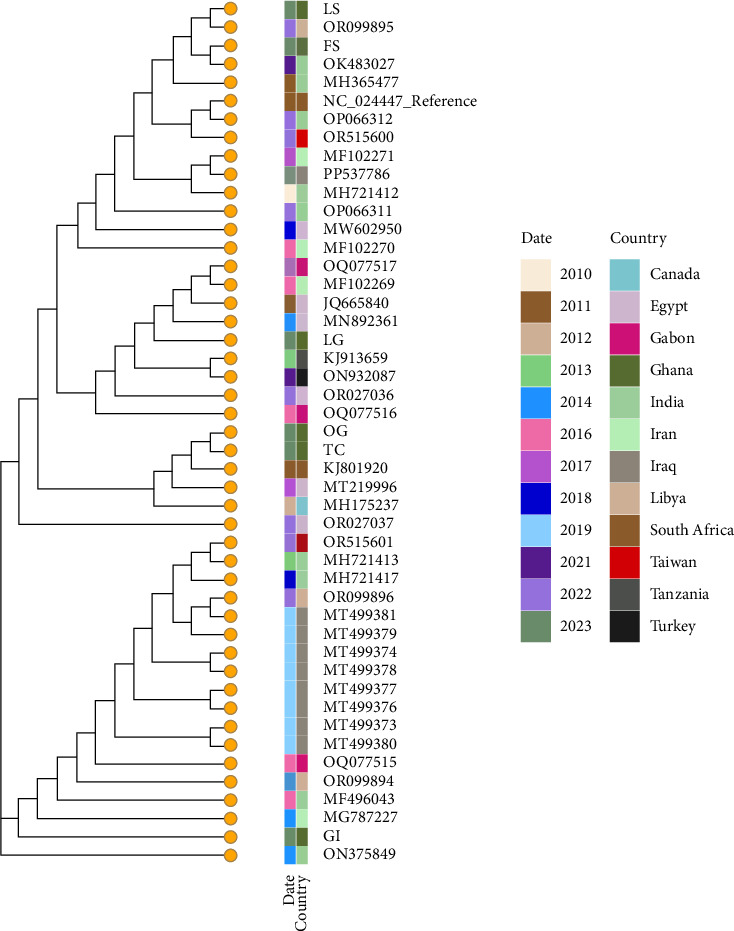
Phylogenetic reconstruction of the pigeon pox virus in Ghana compared to other countries (*n* = 47).

Six samples were sequenced (BioProject accession number PRJNA1298812, Supporting Table 1). They mapped to the reference NC_024447.1 Feral Pigeon pox isolate (FeP2) sequence (> 99% similarity), thus confirming the presence of pigeonpox in the samples (Figure 3). These sequences from Ghana were compared in a phylogenetic framework with 40 representative sequences from various countries and they showed to have > 99% similarity. The reference sequence also had > 99% sequence similarity with a high confidence e-value (Supporting Table 1).

Maximum likelihood phylogenetic tree containing six pigeonpox sequences generated in Ghana and analyzed on the backdrop of the global reference sequence, and 40 highly identical sequences were downloaded from the NCBI database (> 90% blast percent identity; *n* = 47). The terminal nodes are colored to indicate they are all pigeonpox, followed by a heatmap color scheme for date of sample collection and the country of origin. LS: cutaneous lesion (SAMN50285582), OG: esophagus (SAMN50285585), TC: trachea (SAMN50285583), FS: feather stalk (SAMN50285580), GI: gastrointestinal tract (SAMN50285581), and LG: lungs (SAMN50285584). BioProject accession number PRJNA1298812.

## 4. Discussion

This is the first confirmation of PPV in Ghana. The tentative diagnosis was made using observation of clinical signs coupled with the presence of Bollinger bodies, and the hyperkeratosis or hyperplasia of the basal epithelium is diagnostic of avian pox [15, 28]. Definitive diagnosis of PPV was by PCR confirmation as recommended [1, 7, 20, 22, 29]. Even though no specific lesions, as described by Audarya et al. [12], were observed from the CAM, the gel images confirmed the PCR amplification of the P4b gene. An identical observation was reported by Abd El-Samie et al. [1]. The infected pigeon presented with both the cutaneous and diphtheritic forms. While it relatively common for the cutaneous form to occur [2, 6, 7, 11, 12, 21, 25, 30, 31], the diphtheritic form is uncommon and usually associated with young animals [29, 32, 33]. The occurrence of both forms of pigeonpox disease in a bird is rare [17, 34] and usually associated with bacterial infection [35, 36].

There is no known treatment for pigeon pox disease, and attempts at treatment are usually not recommended since it might involve the disruption of the lesions which aids the spread of the virus [2, 37]. Generally, attempts at treating birds with diphtheritic forms of pox are not successful. Wet forms of pigeon pox are complicated with inanition due to lesions in the oral cavity and respiratory tract [9]. The accompanying dysphagia associated with formation of the diphtheritic nodules in the oral cavity makes it impractical to give oral medicines. The combination of the digestive and respiratory factors results in higher mortality in pigeons with wet forms [11]. Euthanasia is recommended in severe cases, most instances of wet forms, and in high-density colony situations as a means of preventing the spread of the disease [2]. The severity of the case, the possibility of spreading to the other birds, and the admittance of the owner not to be able to follow through with the treatment regimen influenced the decision to euthanize the pigeon. Thus, the euthanasia allowed for the disinfection of the premises with the aim of preventing the perpetuation of a rather environmentally stable virus [38].

However, in mild cases, administration of supportive and preventive treatment including disinfection and administration of electrolytes, multivitamin, and antibiotics have been found to reduce mortality [34]. For instance, the use of acyclovir (80 mg/kg QID PO 8–10 days) [10], azithromycin (20 mg/kg BID) [10, 11], or enrofloxacin (10 mg/kg IM for 14 days) [31] in conjunction with vitamin supplementation and topical application of turmeric and neem leaf paste on the cutaneous lesions or liver tonic [10, 11, 14, 39] proved useful in treating the cutaneous form of pigeon pox disease. In situations where the regimens involving antibiotics are preferred, there is the need to ascertain the presence of concomitant infections that might contribute to the pathogenicity of the PPV, especially in carrier animals. This also ensures that veterinarians, in an attempt to treat sick animals, do not contribute to antimicrobial resistance. To ensure the elimination of the viruses from the environment, 1% KOH, 2% NaOH, and 5% phenol have been recommended [9]. This is a biosecurity measure that helps reduce the rate of transmission to naïve birds on the farm or other farms during an epornitic.

The complete relatedness of all the strains to the feral PPV isolate (FeP2) which has been detected globally in countries including South Africa [22, 40, 41], Egypt [33], and India [42] and other PPV isolates in Iraq, Egypt, Gabon, India, Canada, Nigeria, South Africa, and Turkey supports the ubiquity of avipoxviruses [18, 19, 29, 33, 34] and suggests the presence or circulation of PPV in Ghana. The phylogenetic tree shows high similarity and relatedness of the Ghanaian sequences with sequences from other countries, a pattern that could reflect a possible introduction to Ghana. However, more studies are required to ascertain the source of the PPV isolates from Ghana. There is also the need to study the risk to free living wild birds and possible mutations in PPV that might favor spillovers into species not classically considered susceptible to PPV. Increasing evidence supporting the possibility of PPV breaking the species barrier [6, 18, 32, 42–45], as opposed to being species-specific [7, 33, 41, 46–48], favors the need for such studies. Hence, while this could be an isolated case, it highlights the need for active surveillance in determining the distribution and potential hazard of the PPV viruses to pigeons and other avian species of production significant to Ghana. Such surveillance can be conducted in aviaries and poultry farms that have pigeons as it will provide insight into both the distribution of the virus in pigeons and the probability for cross-species transmission.

## Data Availability

The data that support the findings of this study are available from the corresponding author upon reasonable request.

## Supporting Information

Supporting Information 1 Supporting Figure 1 shows the isolation of pigeonpox virus using embryonated eggs.

Supporting Information 2 Supporting Figure 2 shows the agarose gel electrophoresis of the PCR product analysis of the P4b gene amplified from the isolated viruses.

Supporting Information 3 Supporting Table 1 shows the metadata for the selected PPV sequences (including the isolated strains) used for the phylogenetic analysis.

## References

[1] Abd El-Samie H. A., Mohamed H. S., Al-Bakry I., Manal A. (2015). Genomic Characterisation of Pigeon Pox Virus in Egypt. *Zagazig Veterinary Journal*.

[2] Hibl B. M., Blackwood R. S., Simons B. W., Collins D. E. (2019). Poxvirus Infection in a Colony of Laboratory Pigeons (*Columba livia*). *Comparative Medicine*.

[3] Radwan A., Mikhael C. (2020). Comparative Evaluation on the Efficacy of Embryonated Chicken Egg Adapted and Tissue Culture Pigeon Pox Vaccines Against the Local Virulent Strain. *Journal of Applied Veterinary Sciences*.

[4] ICTV (2023). Avipoxvirus. *International Committee on Taxonomy of Viruses (ICTV) Report*.

[5] Sultana R., Nazir K. N. H., Rahman M. T. (2019). Isolation and Molecular Detection of Fowl Pox and Pigeon Pox Viruses for the Development of Live Attenuated Vaccine Seeds From the Local Isolates. *Journal of the Bangladesh Agricultural University*.

[6] Khaleefah I. A., Al-Tameemi H. M., Kraidi Q. A., Najem H. A., Ahmed J. A., Alrafas H. R. (2024). Clinical and Molecular Detection of Fowl Pox in Domestic Pigeons in Basrah Southern of Iraq. *Korean Journal of Veterinary Research*.

[7] Kabir L., Haque E., Borty S. C. (2015). Isolation and Molecular Detection of Fowl Pox and Pigeon Pox Viruses From Recent Outbreak in Bangladesh. *Indian Journal of Life Sciences*.

[8] Williams R. A. J., Truchado D. A., Benitez L. (2021). A Review on the Prevalence of Poxvirus Disease in Free-Living and Captive Wild Birds. *Microbiology Research*.

[9] Greenacre C. B. (2005). Viral Diseases of Companion Birds. *Veterinary Clinics of North America: Exotic Animal Practice*.

[10] Somasundaram C., Raj A., Rangasamy A. (2020). Therapeutic Management of Cutaneous Form of Pigeon Pox in Coimbatore District of Tamil Nadu. *International Journal of Livestock Research*.

[11] Reddy B S., Sivajothi S., Reddy B S., S S. (2018). Therapeutic Management of Cutaneous Form of Pox in Pigeons With Azithromycin. *International International Journal of Avian & Wildlife Biology*.

[12] Audarya S. D., Riyesh T., Kumar N. (2018). Molecular Diagnosis of a Cutaneous Form of Pox in Pigeons at Mhow in Madhya Pradesh. *International Journal of Current Microbiology and Applied Sciences*.

[13] Haller S. L., Peng C., McFadden G., Rothenburg S. (2014). Poxviruses and the Evolution of Host Range and Virulence. *Infection, Genetics and Evolution*.

[14] Tripathy D. N., Reed W. M. (2013). Pox. *Diseases of Poultry*.

[15] Dharanesha N. K., Baghel K. R., Pathak M., Saminathan M. (2020). Gross and Histopathology of Avian Pox in a Feral Pigeon (Columba spp)-A Case Report. *Indian Journal of Veterinary Pathology*.

[16] Tudor D. C. (1978). Pigeon Pox: A Continuing Problem.

[17] Akanbi O. B., Rimfa A. G., Okewole P. A. (2016). Comparative Study on Diphtheritic, Cutaneous and Systemic Forms of Natural Avipoxvirus Infection in Chickens. *Journal of World’s Poultry Research*.

[18] Sharma B., Nashiruddullah N., Bhat M. A. (2019). Occurrence and Phylogenetic Analysis of Avipoxvirus Isolated From Birds Around Jammu. *VirusDisease*.

[19] van Riper I. I. I. C., Forrester D., Thomas N. J., Hunter D. B., Atkinson C. T. (2007). Avian Pox. *Infectious Diseases of Wild Birds*.

[20] Abd El Hafez M. S., Shosha E. A. E. M., Ibrahim S. M. (2021). Isolation and Molecular Detection of Pigeon Pox Virusin Assiut and New Valley Governorates. *Journal of Virological Methods*.

[21] Bwala D. G., Fasina F. O., Duncan N. M. (2015). Avian Poxvirus in a Free-Range Juvenile Speckled (Rock) Pigeon (Columba Guinea). *Journal of the South African Veterinary Association*.

[22] Offerman K., Carulei O., van der Walt A. P., Douglass N., Williamson A.-L. (2014). The Complete Genome Sequences of Poxviruses Isolated from a Penguin and a Pigeon in South Africa and Comparison to Other Sequenced Avipoxviruses. *BMC Genomics*.

[23] Ouma E. A., Kankya C., Dione M. (2023). Poultry Health Constraints in Smallholder Village Poultry Systems in Northern Ghana and Central Tanzania. *Frontiers in Veterinary Science*.

[24] Butcher G. D., Miles R. D. (2019). Avian Necropsy Techniques. https://edis.ifas.ufl.edu/publication/VM009.

[25] Rahman S., Islam M., Islam M., Nazir K., Khan M. (2019). Isolation and Molecular Detection of Avipox Virus from Field Outbreaks in Mymensingh, Bangladesh. *Journal of Advanced Veterinary and Animal Research*.

[26] Zhang Z., Schwartz S., Wagner L., Miller W., Miller W. (2000). A Greedy Algorithm for Aligning DNA Sequences. *Journal of Computational Biology*.

[27] Hadfield J., Megill C., Bell S. M. (2018). Nextstrain: Real-Time Tracking of Pathogen Evolution. *Bioinformatics*.

[28] Oie (2018). OIE Terrestial Manual; Fowl Pox. *OIE Terrestial Manual*.

[29] Pandey M., Ghosh R., Behera P. (2014). Detection of Pox Virus in Domestic Pigeon. *Indian Journal of Veterinary Pathology*.

[30] Mohan M., Fernandez T. F. (2008). A Case Report of Pigeon Pox-Histopathologic Diagnosis. *Veterinary World*.

[31] Smriti S., Upasana V., Rs S., Sb M. (2023). Efficacy of Enrofloxacin for the Therapeutic Management of Cutaneous Form of Pigeon Pox. *The Pharma Innovation Journal*.

[32] Hartati S., Untari T., Lulu Nuraini A., Nururrozi A. (2021). A Case Report of Outbreak Avian Pox Virus from Layer Chickens and a Pigeon in Yogyakarta, Indonesia. *Advances in Animal and Veterinary Sciences*.

[33] Lebdah M., Hassanin O., Ali A. (2019). Avipoxvirus in Egypt and African Continent: A Review. *Zagazig Veterinary Journal*.

[34] Wazari M. I., Saidu L. (2022). Cuteneous and Diphtheritic Forms of Avian Pox Infections in a Juvenile Ostrich. *Nigerian Veterinary Journal*.

[35] Chrobak-Chmiel D., Kwiecień E., Golke A. (2021). Pigeons as Carriers of Clinically Relevant Multidrug-Resistant pathogens—A Clinical Case Report and Literature Review. *Frontiers in Veterinary Science*.

[36] Pledger A. (2005). Avian Pox Virus Infection in a Mourning Dove. *Canadian Veterinary Journal*.

[37] Ha H. J., Alley M., Howe L., Gartrell B. (2013). Evaluation of the Pathogenicity of Avipoxvirus Strains Isolated from Wild Birds in New Zealand and the Efficacy of a Fowlpox Vaccine in Passerines. *Veterinary Microbiology*.

[38] Martain A. R. (2023). Avian Poxvirus. https://www.wildlifecenter.org/avian-poxvirus.

[39] Alexander D. J., Calnek B. W., Barnes H. J., Beard C. W., McDougald L. R. (2003). *Diseases of Poultry*.

[40] He L., Zhang Y., Jia Y. (2023). A Novel Pathogenic Avipoxvirus Infecting Oriental Turtle Dove (Streptopelia orientalis) in China Shows a High Genomic and Evolutionary Proximity With the Pigeon Avipoxviruses Isolated Globally. *Microbiology Spectrum*.

[41] Offerman K., Deffur A., Carulei O., Wilkinson R., Douglass N., Williamson A. L. (2015). Six Host-Range Restricted Poxviruses From Three Genera Induce Distinct Gene Expression Profiles in an in Vivo Mouse Model. *BMC Genomics*.

[42] Pravas Sahu B. (2023). First Complete Genome Characterization of an Indian Pigeon Pox Virus Directly 1 From a Clinical Sample. *BioRix Prepr*.

[43] Abdallah F. M., Hassanin O. (2013). Detection and Molecular Characterization of Avipoxviruses Isolated from Different Avian Species in Egypt. *Virus Genes*.

[44] Mohammed S. K. (2005). *Pathogenic, Antigenic and Serological Relationship Between Fowl Pox, Pigeon Pox and Canary Pox Viruses*.

[45] Boosinger T. R., Winterfield R. W., Feldman D. S., Dhillon A. S. (1982). Psittacine Pox Virus: Virus Isolation and Identification, Transmission, and cross-challenge Studies in Parrots and Chickens. *Avian Diseases*.

[46] Weli S. C., Traavik T., Tryland M., Coucheron D. H., Nilssen Ø. (2004). Analysis and Comparison of the 4b Core Protein Gene of Avipoxviruses From Wild Birds: Evidence for Interspecies Spatial Phylogenetic Variation. *Archives of Virology*.

[47] Mosad S., El-Tholoth M., El-Kenawy A. (2019). Conventional and Molecular Detection of Avipoxviruses From Chickens, Pigeons and Turkeys. *Mansoura Veterinary Medical Journal*.

[48] Sharma B., Nashiruddullah N., Ahmed J. A., Sharma S., Ahamad D. B. (2019). Host Susceptibility of Different Avipoxvirus Isolates. *Journal of Entomology and Zoology Studies*.

[49] Abbiw R. K., Amoako K. K., Enyetornye B. (2024). Isolation and Molecular Detection of Pigeon Pox Virus in a Pigeon With Both Cutaneous and Diphtheritic Forms of Pigeon Pox in Ghana.

