# Supplementary material for: Isolation and Molecular Detection of Pigeonpox Virus in a Pigeon With Both Cutaneous and Diphtheritic Forms of Pigeon Pox Disease in Ghana
Source: Vet Med Int. 2025 Oct 26;2025:7523480. doi: 10.1155/vmi/7523480 (PMC12580035; doi:10.1155/vmi/7523480)
Supplement: Supporting Information 1 — Supporting Figure 1 shows the isolation of pigeonpox virus using embryonated eggs. [file 7523480.f1.docx]

Supplementary Figure 1


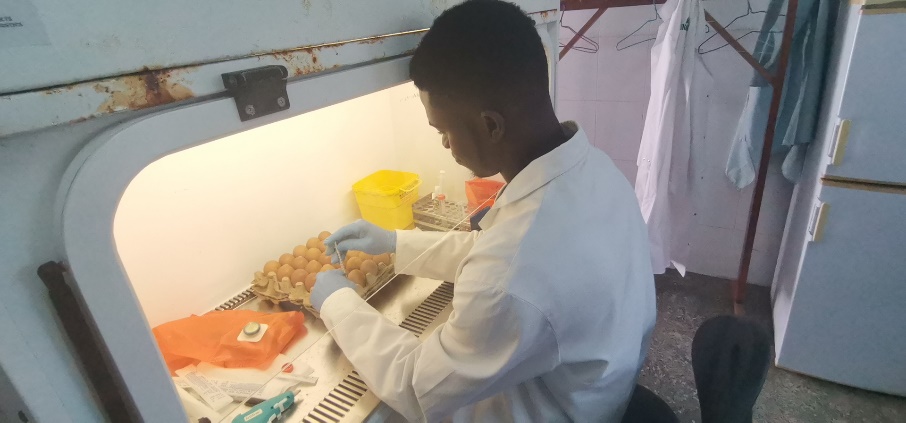

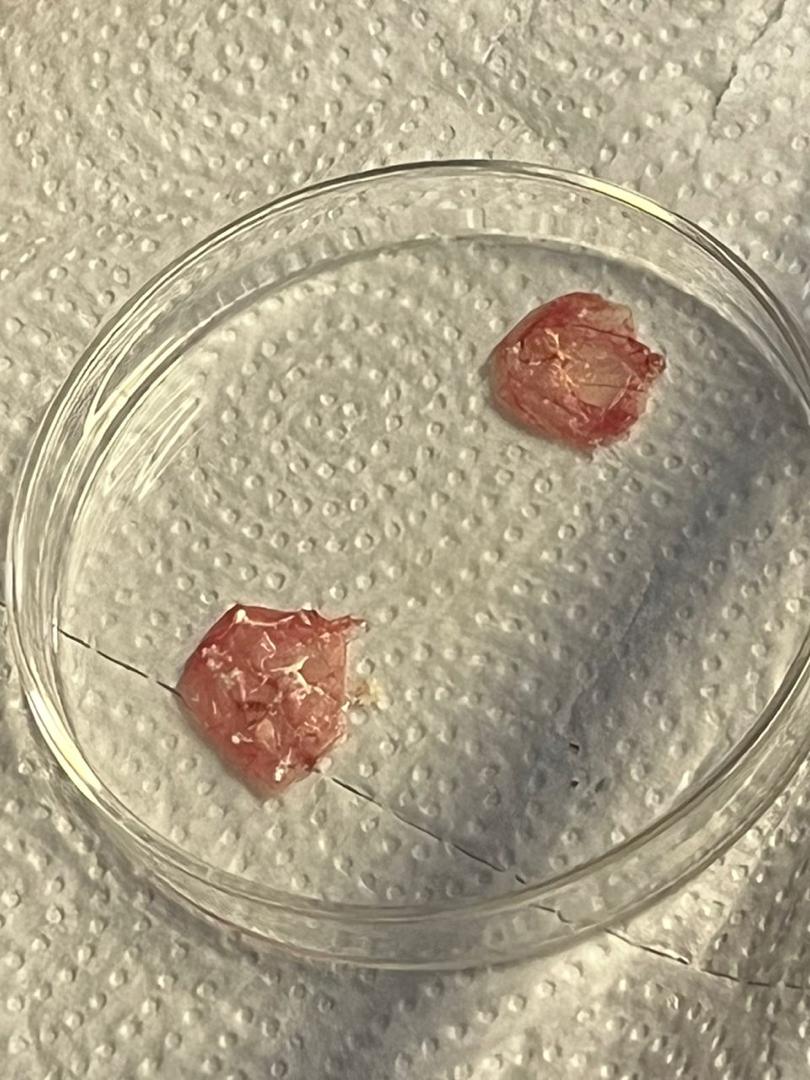


[Grab your reader’s attention with a great quote from the document or use this space to emphasize a key point. To place this text box anywhere on the page, just drag it.]

**B**

**A**

Supplementary 1: Isolation of pigeon pox virus using the embryonated eggs.

**A.** shows the preparation of the inoculum and **B.** shows the harvested chorioallantoic membrane (CAM). Even though no specific lesions, as described by Audarya et al.^[[1]](#footnote-1)^, where observed from the CAM, the gel images confirmed the PCR amplification of the P4b gene. An identical observation was reported by Abd El-Samie et al.^[[2]](#footnote-2)^.

1. S.D. Audarya and others, ‘Molecular Diagnosis of a Cutaneous Form of Pox in Pigeons at Mhow in Madhya Pradesh’, *International Journal of Current Microbiology and Applied Sciences*, 7.09 (2018), 1318–23 <https://doi.org/10.20546/ijcmas.2018.709.157>. [↑](#footnote-ref-1)
2. H A Abd El-Samie and others, ‘Genomic Characterisation of Pigeon Pox Virus in Egypt’, *Zagazig Veterinary Journal*, 43.1 (2015), 94–101. [↑](#footnote-ref-2)
