# Supplementary material for: Isolation and Molecular Detection of Pigeonpox Virus in a Pigeon With Both Cutaneous and Diphtheritic Forms of Pigeon Pox Disease in Ghana
Source: Vet Med Int. 2025 Oct 26;2025:7523480. doi: 10.1155/vmi/7523480 (PMC12580035; doi:10.1155/vmi/7523480)
Supplement: Supporting Information 2 — Supporting Figure 2 shows the agarose gel electrophoresis of the PCR product analysis of the P4b gene amplified from the isolated viruses. [file 7523480.f2.docx]

Supplementary Figure 2


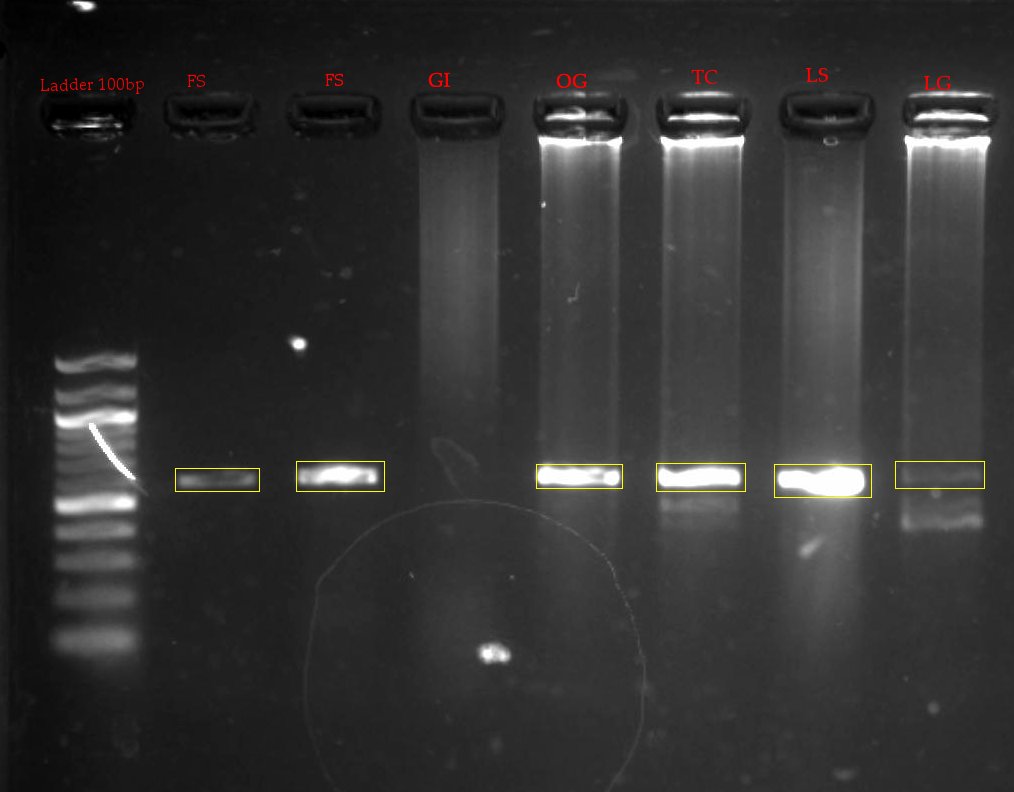


100

500

600

Supplementary 3: Agarose gel electrophoresis of the PCR products analysis of the P4b gene amplified from the isolated viruses.

FS -feathered stock, GI- Gastrointestinal tract, OG- Oesophagus, TC- Trachea, LS- cutaneous lesions and LG- Lungs
