# Supplementary material for: Isolation and Molecular Detection of Pigeonpox Virus in a Pigeon With Both Cutaneous and Diphtheritic Forms of Pigeon Pox Disease in Ghana
Source: Vet Med Int. 2025 Oct 26;2025:7523480. doi: 10.1155/vmi/7523480 (PMC12580035; doi:10.1155/vmi/7523480)
Supplement: Supporting Information 3 — Supporting Table 1 shows the metadata for the selected PPV sequences (including the isolated strains) used for the phylogenetic analysis. [file 7523480.f3.docx]

Supplementary Table 1

Table 1: Meta data for selected PPV 47 sequences

| **ID** | **Accession** | **Country** | **Collection Date** | **Description** | **Total Score** | **Query Cover** | **E value** | **Per. ident** |
| --- | --- | --- | --- | --- | --- | --- | --- | --- |
| MH175237 | MH175237.1 | Canada | 2012 | Pigeonpox virus isolate ROPI/W370/ON/2012 4b virion core protein (4b) gene, partial cds | 979 | 90% | 0 | 100 |
| OR027036 | OR027036.1 | Egypt | 2022 | Pigeonpox virus isolate PP/EG/SHARQIA-6 P4B gene, partial cds | 974 | 90% | 0 | 100 |
| OQ077515 | OQ077515.1 | Gabon | 2016 | Pigeonpox virus isolate ow_2016_1 4b core protein gene, partial cds | 1029 | 95% | 0 | 100 |
| ON375849 | ON375849.1 | India | 2014 | Pigeonpox virus isolate PPV/Pur-Od-4b/01/Ind, complete genome | 1077 | 100% | 0 | 100 |
| MF496043 | MF496043.1 | India | 2016 | Pigeonpox virus major core protein P4b gene, partial cds | 926 | 85% | 0 | 100 |
| MH721412 | MH721412.1 | India | 2010 | Pigeonpox virus isolate PPV/Dhe-Od-4b/01 P4B core protein (4b) gene, partial cds | 904 | 83% | 0 | 100 |
| MH365477 | MH365477.1 | India | 2011 | Pigeonpox virus isolate PGPV1 P4b core protein (P4b) gene, partial cds | 891 | 82% | 0 | 100 |
| OP066312 | OP066312.1 | India | 2022 | Pigeonpox virus isolate SVPUAT_Meerut_03 P4b core protein (P4b) gene, partial cds | 867 | 80% | 0 | 100 |
| OP066311 | OP066311.1 | India | 2022 | Pigeonpox virus isolate SVPUAT_Meerut_02 P4b core protein (P4b) gene, partial cds | 837 | 77% | 0 | 100 |
| MF102269 | MF102269.1 | Iran | 2016 | Pigeonpox virus isolate FZRP7C 4b core protein (P4b) gene, partial cds | 990 | 91% | 0 | 100 |
| MG787227 | MG787227.1 | Iran | 2014 | Pigeonpox virus isolate PiPVIR18 P4b (p4b) gene, partial cds | 926 | 85% | 0 | 100 |
| PP537786 | PP537786.1 | Iraq | 2023 | Pigeonpox virus isolate Khitam-W19 virion core protein P4b (P4b) gene, partial cds | 1074 | 99% | 0 | 100 |
| KJ801920 | KJ801920.1 | South Africa | 2011 | Pigeonpox virus isolate FeP2, complete genome | 1077 | 100% | 0 | 100 |
| NC_024447_Ref | NC_024447.1 | South Africa | 2011 | Pigeonpox virus isolate FeP2, complete genome | 1077 | 100% | 0 | 100 |
| OR515600 | OR515600.1 | Taiwan | 2022 | Pigeonpox virus isolate Taichung p4b gene, partial cds | 928 | 86% | 0 | 100 |
| OQ077517 | OQ077517.1 | Gabon | 2017 | Pigeonpox virus isolate ow_2017_3 4b core protein gene, partial cds | 1024 | 95% | 0 | 99.82 |
| MF102270 | MF102270.1 | Iran | 2016 | Pigeonpox virus isolate FZRP9C 4b core protein (P4b) gene, partial cds | 996 | 92% | 0 | 99.82 |
| OR027037 | OR027037.1 | Egypt | 2022 | Pigeonpox virus isolate PP/EG/SHARQIA-7 P4B gene, partial cds | 979 | 91% | 0 | 99.81 |
| MT219996 | MT219996.1 | Egypt | 2017 | Pigeonpox virus strain mans17P P4b core protein gene, partial cds | 974 | 90% | 0 | 99.81 |
| MN892361 | MN892361.1 | Egypt | 2014 | Pigeonpox virus isolate PPLH 4b gene, partial cds | 950 | 88% | 0 | 99.81 |
| MT499377 | MT499377.1 | Iraq | 2019 | Pigeonpox virus isolate RSJ6 4b core protein (p4b) gene, partial cds | 760 | 71% | 0 | 99.76 |
| MT499380 | MT499380.1 | Iraq | 2019 | Pigeonpox virus isolate RSJ9 4b core protein (p4b) gene, partial cds | 760 | 71% | 0 | 99.76 |
| OQ077516 | OQ077516.1 | Gabon | 2016 | Pigeonpox virus isolate ow_2016_2 4b core protein gene, partial cds | 1057 | 99% | 0 | 99.65 |
| OK483027 | OK483027.1 | India | 2021 | Pigeonpox virus isolate PPF_2 4b core protein gene, partial cds | 1053 | 98% | 0 | 99.65 |
| JQ665840 | JQ665840.1 | Egypt | 2011 | Pigeonpox virus strain ELsharqyia_PGPV 4b virion core protein gene, partial cds | 1026 | 96% | 0 | 99.64 |
| MF102271 | MF102271.1 | Iran | 2017 | Pigeonpox virus isolate FZRP5C 4b core protein (P4b) gene, partial cds | 970 | 91% | 0 | 99.62 |
| MW602950 | MW602950.1 | Egypt | 2018 | Pigeonpox virus isolate PPVNV1 P4b (4b) gene, partial cds | 933 | 87% | 0 | 99.61 |
| OR099896 | OR099896.1 | Libya | 2022 | Pigeonpox virus strain LW core protein P4b gene, partial cds | 931 | 87% | 0 | 99.61 |
| MH721417 | MH721417.1 | India | 2018 | Pigeonpox virus isolate PPV/Nab-Od-4b/01 P4B core protein (4b) gene, partial cds | 893 | 83% | 0 | 99.59 |
| MT499373 | MT499373.1 | Iraq | 2019 | Pigeonpox virus isolate RSJ2 4b core protein (p4b) gene, partial cds | 754 | 71% | 0 | 99.52 |
| MT499376 | MT499376.1 | Iraq | 2019 | Pigeonpox virus isolate RSJ5 4b core protein (p4b) gene, partial cds | 754 | 71% | 0 | 99.52 |
| MT499381 | MT499381.1 | Iraq | 2019 | Pigeonpox virus isolate RSJ10 4b core protein (p4b) gene, partial cds | 754 | 71% | 0 | 99.52 |
| KJ913659 | KJ913659.1 | Tanzania | 2013 | Pigeonpox virus isolate PGPV-TZ P4b core protein (P4b) gene, partial cds | 1040 | 98% | 0 | 99.48 |
| OR099895 | OR099895.1 | Libya | 2022 | Pigeonpox virus strain Li core protein P4b gene, partial cds | 926 | 87% | 0 | 99.41 |
| MH721413 | MH721413.1 | India | 2013 | Pigeonpox virus isolate PPV/Bha-Od-4b/01 P4B core protein (4b) gene, partial cds | 887 | 83% | 0 | 99.39 |
| ON932087 | ON932087.1 | Turkey | 2021 | Pigeonpox virus isolate PPV/Harran/13 virion core protein P4b (HM89_gp173) gene, partial cds | 1051 | 99% | 0 | 99.31 |
| MT499374 | MT499374.1 | Iraq | 2019 | Pigeonpox virus isolate RSJ3 4b core protein (p4b) gene, partial cds | 749 | 71% | 0 | 99.28 |
| MT499378 | MT499378.1 | Iraq | 2019 | Pigeonpox virus isolate RSJ7 4b core protein (p4b) gene, partial cds | 749 | 71% | 0 | 99.28 |
| MT499379 | MT499379.1 | Iraq | 2019 | Pigeonpox virus isolate RSJ8 4b core protein (p4b) gene, partial cds | 749 | 71% | 0 | 99.28 |
| OR099894 | OR099894.1 | Libya | 2014 | Pigeonpox virus strain LP core protein P4b gene, partial cds | 920 | 87% | 0 | 99.22 |
| OR515601 | OR515601.1 | Taiwan | 2022 | Pigeonpox virus isolate Taichung p4b gene, partial cds | 689 | 86% | 0 | 91.43 |
| FS |  | Ghana | 2023 |  |  |  |  |  |
| GI |  | Ghana | 2023 |  |  |  |  |  |
| LG |  | Ghana | 2023 |  |  |  |  |  |
| LS |  | Ghana | 2023 |  |  |  |  |  |
| OG |  | Ghana | 2023 |  |  |  |  |  |
| TC |  | Ghana | 2023 |  |  |  |  |  |
